# Selecting for infectivity across metapopulations can increase virulence in the social microbe *Bacillus thuringiensis*

**DOI:** 10.1111/eva.13529

**Published:** 2023-01-16

**Authors:** Tatiana Dimitriu, Wided Souissi, Peter Morwool, Alistair Darby, Neil Crickmore, Ben Raymond

**Affiliations:** ^1^ Centre for Ecology and Conservation University of Exeter Penryn UK; ^2^ School of Life Sciences University of Sussex Brighton UK; ^3^ Centre for Genomic Research, Institute of Integrative Biology University of Liverpool Liverpool UK

**Keywords:** *Bacillus thuringiensis*, directed evolution, evolution of virulence, mutators, public goods, social evolution

## Abstract

Passage experiments that sequentially infect hosts with parasites have long been used to manipulate virulence. However, for many invertebrate pathogens, passage has been applied naively without a full theoretical understanding of how best to select for increased virulence and this has led to very mixed results. Understanding the evolution of virulence is complex because selection on parasites occurs across multiple spatial scales with potentially different conflicts operating on parasites with different life histories. For example, in social microbes, strong selection on replication rate within hosts can lead to cheating and loss of virulence, because investment in public goods virulence reduces replication rate. In this study, we tested how varying mutation supply and selection for infectivity or pathogen yield (population size in hosts) affected the evolution of virulence against resistant hosts in the specialist insect pathogen *Bacillus thuringiensis*, aiming to optimize methods for strain improvement against a difficult to kill insect target. We show that selection for infectivity using competition between subpopulations in a metapopulation prevents social cheating, acts to retain key virulence plasmids, and facilitates increased virulence. Increased virulence was associated with reduced efficiency of sporulation, and possible loss of function in putative regulatory genes but not with altered expression of the primary virulence factors. Selection in a metapopulation provides a broadly applicable tool for improving the efficacy of biocontrol agents. Moreover, a structured host population can facilitate artificial selection on infectivity, while selection on life‐history traits such as faster replication or larger population sizes can reduce virulence in social microbes.

## 1 INTRODUCTION

Passage, the repeated infection and re‐isolation of a microbe in a host, has been used as a tool for the manipulation of parasite virulence for decades, as well as a means of testing evolution of virulence theory (Ebert, 1998; Raymond & Erdos, 2022). Passage has been used as a means of producing attenuated live vaccines: sequential infection of animal tissue cultures can lead to loss of virulence in human hosts and has been used to produce polio and yellow fever vaccines among others (Barrett, 2017; Sabin & Boulger, 1973). Conversely, adaptation of viruses to a particular host via passage can lead to increased virulence (Ebert, 1998). Although there are complex relationships between virulence and fitness in natural populations (Alizon et al., 2009; Frank, 1996; Gandon et al., 2001), artificial inoculation means that many of the negative consequences of high virulence in terms of reducing opportunities for transmission will not operate in laboratory conditions and so it may be possible to increase the virulence of naturally occurring pathogens via artificial selection.

Increasing pathogen virulence via passage has long been a goal in biocontrol research as researchers have sought to manipulate the efficacy and/or host range of microbes that are potential biocontrol agents (Raymond & Erdos, 2022). Naïve passage designs, in which the aim is simple infection and re‐infection, without any other selection pressure or modifications, can increase the virulence of baculoviruses in terms of reducing doses required to kill 50% of insects (LD_50_; Berling et al., 2009; Kolodny‐Hirsch & Van Beek, 1997; Maleki‐Milani, 1978). Simple passage of baculoviruses can increase virulence partly because of the high genetic diversity found in natural populations (Shapiro et al., 1992; Thézé et al., 2014). However, another reason naïve passage can be effective is if reproductive rate within hosts is positively correlated with virulence, as is assumed by many classical models of virulence (Alizon et al., 2009; Frank, 1996; Gandon et al., 2001). Even in the simplest passage design, there will be competition between genetic variants within hosts, and we would expect that this within‐host competition would favor higher replication rate, ignoring issues such as antagonism and evasion or manipulation of immunity (Massey et al., 2004; Raberg et al., 2006). This means that simple isolation and re‐infection can select for the genotypes which grow faster within hosts which can produce the net result of an increase in virulence without the need for any additional selection pressure.

Nevertheless, research on the social biology of microorganisms emphasizes that increased replication rate does not necessarily lead to increased virulence (Buckling & Brockhurst, 2008; Raymond & Erdos, 2022). Many bacterial pathogens, for instance, invest considerable resources in producing virulence factors such as toxins or siderophores that are important for infecting hosts or accessing host resources such as iron (Diard, Garcia, et al., 2014; Raymond et al., 2012; West & Buckling, 2003). Investing in these resources can slow replication rates, this means that intense within‐host competition can select for cheaters which can outcompete virulent genotypes in mixed infections, although cheaters are expected to have low fitness in clonal infections (Granato et al., 2018; Pollitt et al., 2014; Rumbaugh et al., 2012). In addition, increased genetic diversity within infections (low relatedness) will also tend to favor fast‐replicating genotypes such as cheaters. These social biology concepts are relevant both for clinically important microbes and for biocontrol agents. Experimental evolution with entomopathogenic nematodes, for instance, shows increasing opportunities for cheating can lead to attenuation and extinction and may explain historical problems with unstable virulence in laboratory culture of these parasites (Shapiro‐Ilan & Raymond, 2016).

In this study, we aimed to apply social evolution theory to increase the virulence of the Gram‐positive invertebrate pathogen, *Bacillus thuringiensis* (Bt). Bt is a valuable model for testing novel passage regimes partly because its virulence factors are extremely well characterized (Adang et al., 2014) and because its social biology is well studied (Cornforth et al., 2015; Raymond et al., 2012; van Leeuwen et al., 2015; Zhou et al., 2014). Bt obligately requires pore‐forming toxins for infection. These are produced in the form of crystalline bodies at sporulation in the cadaver but solubilized in the midgut after ingestion; the toxins disintegrate the midgut epithelium and allow these microbes access to the hemocoel (Adang et al., 2014). In some insect hosts, the production of quorum‐regulated phospholipases and cellulases complements the action of the crystal toxins and facilitates host invasion (Salamitou et al., 2000; Zhou et al., 2014). A range of other virulence factors, for example, vegetative insecticidal proteins (Vips), are produced by most Bt genotypes, although their contribution to pathogenicity is less clear (Chakroun et al., 2016).

**FIGURE 1:**
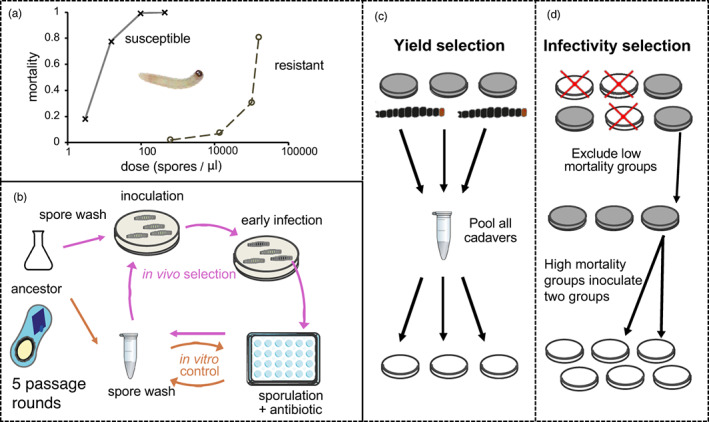
Experimental design to increase the virulence of *Bacillus thuringiensis* against resistant insects. (a) Bioassays using the Bt ancestor 7.1.o show substantial differences in mortality between our focal Bt‐resistant insect VL‐FR and its near‐isogenic Bt susceptible counterpart VL‐SS (*F*_1,5_ *=* 34.1, *p* = 0.0021). The LC50 of the susceptible insects was estimated at 7.4 CFU/μl (95% confidence limits 6.9–7.9 CFU/μl), while the LC50 of the VL‐FR resistant was 5 × 10^5^ CFU/μl (Table S1). Selection experiments began with a dose that would kill 20%–30% of VL‐FR insects. (b) Selection cycles performed separately for strains with mutator or wild‐type mutation rates. For in vivo selection treatments, cells selected from killed larvae were inoculated into sporulation medium. In vitro growth, sporulation, and purification steps were common to the in vivo selection and in vitro control. To prevent the invasion of cheaters not contributing to cooperative virulence, two in vivo selection treatments were compared. (c) In the yield selection treatment, all cadavers from a replicate lineage were pooled and inoculated together for spore production. (d) Under infectivity selection, half of the subpopulations, those causing the lowest mortality, are terminated at the end of each round of selection, while spores from the remaining subpopulations are divided and used to infect two subpopulations in the next round of infection.

The entomocidal toxins of Bt have high selective potency against insect pests and this is the main reason why Bt dominates the microbial biocontrol market and supplies the vast majority of insecticidal toxins for genetically modified crops (Bravo et al., 2011). There is considerable interest in identifying mutants or proteins that can overcome host resistance in Bt (Badran et al., 2016). From a theoretical point of view, it may also make sense to target passage experiments at hosts that are resistant but still vulnerable to some level of infection. Based on fitness landscape theory, we expected that rapid, experimentally tractable evolution would be more likely in a pathogen of low fitness rather than one already at an adaptive peak (Poelwijk et al., 2007). We used a host species commonly targeted by Bt products: the diamondback moth *Plutella xylostella*, using a well‐characterized genotype with a high level of resistance to *B. thuringiensis kurstaki* (Figure 1a). Moreover, for social microbes, the selection pressure acting to maintain virulence does not come from within‐host competition for rapid growth—it has to come from competition between populations in terms of total population size (yield; Griffin et al., 2004) or number of hosts infected (Raymond et al., 2012). This is because the fitness benefits (or public goods) provided by cooperation in known social pathogens either increase the efficient use of host resource or, in the case of the crystal toxins, provide access to host tissues.

In this study, we conducted experimental evolution using cycles of Bt infection in resistant hosts alternating with spore production in vitro (Figure 1b). We tested passage regimes that would maximize selection based on yield (population size within hosts) or infectivity. Selecting for yield involved combining all cadavers from a replicate into a single inoculum pool at each passage, so that genotypes with higher yield are better represented in the next round of infections (Griffin et al., 2004; Shapiro‐Ilan & Raymond, 2016; Figure 1c). This type of between‐host competition has previously produced modest increases in virulence in Bt (Garbutt et al., 2011). Pooling subpopulations also mimic a high pathogen dispersal rate within natural populations (Kümmerli et al., 2009), which is plausible for *Bacillus* (Pearce et al., 2009). The second selection regime involved directly selecting for infectivity. To do this, we imposed competition between subpopulations within a metapopulation (each metapopulation constituting an independent replicate), so that only the 50% most infectious subpopulations are used to initiate the next round of infection (Figure 1d). The most infectious subpopulations are divided and initiate two new pathogen subpopulations in the next round of infection. The infectivity selection treatment, therefore, uses a form of budding dispersal and so may have additional benefits in terms of preserving population structure (Gardner & West, 2006; Kümmerli et al., 2009). It should be emphasized that these selection treatments are not exclusive or perfect; in other words, we cannot prevent some selection for infectivity in the yield treatment (infections have to happen) nor some selection for yield in the infectivity treatment (bacteria have to grow in cadavers), but we are attempting to maximize different types of selection with our experimental treatments.

Finally, we tested how increasing the mutation supply would affect virulence evolution, a common approach in directed evolution (Selifonova et al., 2001). Bacterial clones with elevated mutation rates and defective proofreading genes, or mutators, are prevalent in pathogenic species (Taddei et al., 1997), and we increased mutation supply by approximately 25‐fold by carrying out selection in a mutator, made by disruption of the *mutS* gene, and in lineages using the wild‐type ancestor. At the end of passage experiments, we bio‐assayed for changes in virulence in evolved lineages (diverse independently evolved populations), as well as clones isolated from those lineages. We also measured changes in life history (spore production in vitro, competitive fitness) and tested whether social conflicts might have affected experimental outcomes. Finally, in order to explore the possible mechanisms for the observed increase in virulence in evolved clones, we explored if changes in the expression of known virulence factors could explain results and conducted whole genome sequencing on a selection of evolved clones.

## 2 METHODS

### 2.1 Insects and insect rearing

The Cry1Ac‐resistant line “VL‐FR” of the diamondback moth *P. xylostella* used for experiments was previously derived from a cross of line NOQA‐GE with the diet adapted susceptible line Vero Beach: This line was reared and selected for resistance to Cry1Ac as described previously (Zhou et al., 2018) and can survive exposure to ~10^4^‐fold higher doses of Bt compared to the susceptible line (Figure 1a). We established a population that was fixed for resistance using a PCR screen of resistance alleles in the parental population. An insect strain with similar genetic background (also derived from a NOQA‐GE X Vero Beach cross) was established from F2 offspring but in this case fixed for susceptibility to Cry1Ac, this line was denoted “VL‐SS” (Zhou et al., 2018). Diamondback moth larvae used for selection were reared on autoclaved artificial diet (Baxter et al., 2011) without supplementary antibiotics until third instar. All insects were reared in a controlled temperature facility at the University of Exeter's Cornwall campus.

### 2.2 Bacteria, plasmids, strain construction, and in vitro growth conditions

The ancestor *Bt kurstaki* strain was obtained by transforming the original Bt 7.1.o field isolate (Raymond et al., 2009) with the pHT315‐gfp plasmid containing the *ermB* gene conferring erythromycin (Erm) resistance (Zhou et al., 2014). The mutator (mut) strain was obtained by inactivating the *mutS* gene in 7.1.o via disruption. The *cat* gene conferring chloramphenicol (Chl) resistance was digested from the pAB2 plasmid using KpnI (Bravo et al., 1996); this fragment was inserted at position 1439 of the *mutS*‐coding sequence. The sequence between positions 601 and 1837 of *mutS* gene coding sequence with the *cat* insert was synthesized in pUC57 from GenScript after adding *BamHI* sites on both ends. This synthesized *BamHI* fragment was cloned into the thermosensitive vector pRN1501 (Lereclus et al., 1992). The resulting plasmid was introduced into Bt 7.1.o, and clones with recombination of the interrupted *mutS* sequence into the chromosome were obtained after growth at 42°C by screening for Chl resistance and loss of Erm resistance (Lereclus et al., 1992). Insertion was confirmed by PCR and sequencing of *mutS* locus. The mutation rate of the ancestor with wild‐type *mutS* (wt) and mut strain was calculated with fluctuation assays. Thirty‐two independent cultures per strain were inoculated into sporulation medium by 10^7^‐fold dilution from Luria Broth (LB) overnight cultures. After 24 h, cultures were plated on LB agar (LBA) plates containing 100 μg/ml rifampicin. Mutation rates and confidence intervals were calculated with the Ma‐Sandri‐Sarkar (MSS)‐maximum likelihood method using FALCOR (Hall et al., 2009).

Spores and crystals of *Bacillus thuringiensis* (Bt) for use in selection experiments or bioassays were grown in sporulation media (HCO; Lecadet et al., 1980) containing polymyxin (100 IU/ml) (Oxoid) for 72 h at 30°C with shaking at 150 rpm. Selective antibiotics (10 μg/ml Erm for wt; 5 μg/ml Chl for mut strain) was used in all cultures except for the production of spores for bioassays which used antibiotic free culture. When grown from insect cadavers, 30 μg/ml Erm was used to inhibit growth of Gram‐negative bacteria. Standard spore production conditions used 1 ml of HCO in 24‐well growth plates. Rows of inoculated wells from the same line of selection were alternated with empty rows to prevent and control for contamination between lines. Initial cultures of ancestors at passage 1 used 100 ml cultures in 500 ml flasks. All other routine culture of Bt used overnight growth in LB or on LBA plates at 30°C with antibiotic selection unless otherwise stated.

### 2.3 Passage experiment

Selection was performed with four replicate lineages per strain for each treatment combination, using a factorial design with two strains with diffferent mutation rates (wt and mut) and three treatments (yield, infectivity, and in vitro control), as detailed below. In total, five rounds of selection were performed.

For infection, sterile diet was cut into quarters in 55 ml dishes, and each quarter was inoculated with 90 μl of spore suspension containing 5 × 10^4^ to 10^5^ spores/μl (chosen to produce 25%–30% mortality over 2–4 days for resistant diamondback moth, Figure 1a) and dried. Ten third‐instar VL‐FR larvae were then added per 55 ml dish. Insect death was recorded daily from 2 days after infection. When total mortality reached 25%–30%, the earliest cadavers were transferred to microcentrifuge tubes with sterile toothpicks (Figure 1b). After 2 days at room temperature, 0.85% NaCl solution (saline) was added to the tubes. Cadavers were homogenized with pellet pestles, briefly vortexed and the suspension was transferred to sporulation media (Figure 1b). When growth was complete, 200‐μl spore aliquots were transferred to 96‐well PCR plates with aluminum sealing film for quantification, infection, and storage. Plates were centrifuged at 3000 *g* for 15 min, the supernatant was removed, and spores were resuspended in 100 μl 0.85% NaCl. Plates containing spores to be used in next infection step were pasteurized (20 min, 65°C) and kept at 10°C until use, the others were stored at −20°C. Pasteurization prevents contamination from gut microbes and also selects against fast‐growing asporogenic mutants. Spore density was estimated by measuring OD600 nm in a 96‐well microplate reader of 10‐fold dilutions. A subset of the samples was plated at appropriate dilutions, in order to visually check for contamination and calibrate OD600 nm measurements.

Selection of cadavers and inoculation into sporulation medium depended on experimental treatment (Figure 1). The in vitro control selection treatment culture and washing steps were performed as above but without infection of insects (Figure 1b) and was similarly performed on four lineages per strain (wt and mut) with five rounds of selection. Approximately 5 × 10^4^ spores were used to inoculate 1 ml sporulation medium and cultured as above. After standard culture, spores were centrifuged and resuspended in 0.85% NaCl, pasteurized and kept at 10°C until use.

For the yield selection treatment, each lineage was split into six insect subpopulations using six dishes, which were pooled together at each round of selection. Cadavers from all six subpopulations in the pooled treatment were transferred to the same microfuge tube. Then, 300 μl saline was added and the suspension was distributed into six wells for inoculation. For replicate lineages with more than 30% death across insect populations at time of selection, the number of cadavers taken from subpopulations with most deaths was reduced in order to select 30% cadavers overall and maintain comparable population sizes across treatments at inoculation (Figure 1c).

For the infectivity treatment, each lineage was split into 12 insect subpopulations maintained separately in 12 dishes. The six subpopulations with highest larval mortality were selected and the six other subpopulations were discarded (Figure 1d). If the same mortality was observed for several populations, the ones with earliest observed deaths were selected. A maximum of three cadavers from selected subpopulations were transferred into separate tubes (6 total), then 50 μl saline was added to each tube, and the suspension from each tube was inoculated into separate wells. After spore purification, spores from each well were used to inoculate two subpopulations in the next selection round (Figure 1d).

### 2.4 Phenotypic and genotypic characterization of evolved bacteria

#### 2.4.1 Bioassays

**FIGURE 2:**
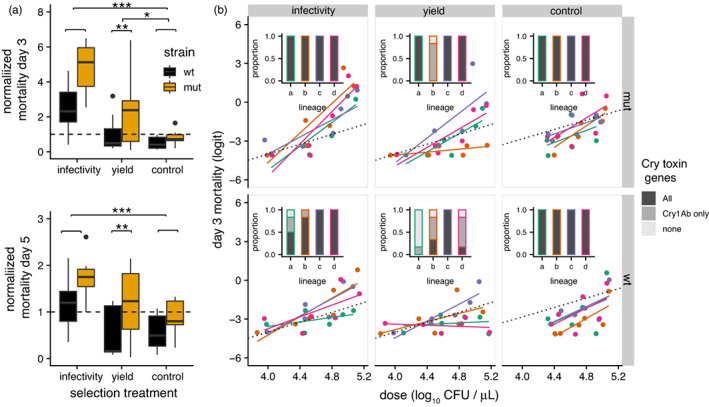
Mutation rate and infectivity selection shape evolution of virulence and retention of public good virulence factors (cry toxins). Data shown are from two separate bioassays performed with independent spore preparations of evolved lineages after five rounds of selection. (a) Proportional mortality (relative to the ancestor) at 3 and 5 days after inoculation for different treatments, control refers to in vitro passaged controls. The center value of the boxplots shows the median, boxes the first and third quartile, and whiskers represent 1.5 times the interquartile range; outliers are shown as dots (*N* = 8, 4 lineages per treatment in two assays). (b) Barplots show the proportion of clones containing no toxin genes (light gray), Cry1Ab only (dark gray), or both Cry1A and Cry2A (black) genes for each lineage (*N* = 6 clones per lineage). Scatter plots show logit‐transformed mortality 3 days after inoculation is shown as a function of Bt dose; lines are fitted models for each lineage. For reference, the dotted black line shows the dose response for the ancestor, the replicate lineages within each treatment are color coded. See also Table S1 and Figures S1 and S2 for results of clone‐level virulence assays.

After five rounds of selection, the evolved lineages were frozen at −80°C (LB 20% glycerol) without pasteurization. These frozen stocks were used to inoculate sporulation medium for assays of evolved lineages. The virulence of each independently evolved lineage (Figure 2) was assayed by scoring proportional mortality (Figure 2) after 3 days (early mortality) and 5 days (late mortality). This analysis used data from two independent bioassays using independently grown spore stocks (minimizing effects due to variation in growth/amplification in vitro). Analyses reported in Figure 2a used average mortality data that were normalized against the ancestor: The average mortality (across the three doses) of each evolved lineage replicate was divided by the average mortality measured in the ancestor within the same assay.

Evolved lineages are genetically diverse populations, which can present challenges when repeating experiments or when attempting to link genotypes to phenotypes. Clones were therefore isolated from lineage samples by streaking single colonies three times on LBA and we conducted bioassays on both clones and lineage after selection was complete. Clone nomenclature in figures and tables indicates strain (*W* for wild‐type mutation rate, *M* for mutator), selection treatment (*y* for yield, *i* for infectivity selected, *v* for in vitro control) with a letter for independent lineage and a number for clone, for example, Wia1, wild type, infectivity selected, lineage a, clone 1.

The viable spore concentration of all cultures used in bioassays was measured by plating after pasteurization and measuring colony forming units (CFUs). Standard virulence bioassays used at least three doses with 60 insects per dose (between 10^4^ and 1.2 × 10^5^ spores/μl for *Bt kurstaki* in resistant *P. xylostella*) and were repeated at least twice. The exception to this was the initial screens of clone level variation of six clones per lineage which used 15–30 insects per dose and two doses. Only strongly melanized cadavers were scored as Bt‐killed insects. The dose response in bioassays of clones was analyzed in terms of viable spores but also in terms of dilution factor of inocula relative to purified spore stocks as some clones displayed variable germination rates. Clones of interest were further characterized in additional assays.

Viable spore counts of clones grown in sporulation media for bioassays were used in analyses of in vitro spore production. Assessment of bacterial development rate used three clones with elevated virulence, three clones with virulence unchanged, as well the wild‐type ancestor. The proportion of cells that were vegetative, the proportion of cells that had toxin crystals within the exosporium and proportion of cells at the final stage of lysis of exosporium were assessed by phase‐contrast microscopy after 72 and 144 h of growth in HCO.

### 2.5 Genetic analysis of Cry toxin gene complements

The ancestor Bt 7.1.o wild type bears genes for several expressed toxins (Cry1Ac, Cry2Aa, Cry1Aa) on a large plasmid (homologous to CP009999, 317 kb) and one additional toxin gene (Cry1Ab) on a smaller plasmid (homologous to CP010003, 69 kb; Tables S2 and S3; Méric et al., 2018). Loss of each of those plasmids was detected in several clones with whole‐genome sequencing. We extended those results by estimating Cry toxin plasmid loss for all clones from evolved lines by PCR (six clones from each insect‐evolved lineage and two clones from each in vitro lineage). Primers were designed and validated with the sequenced clones. Primers Cry2‐F and Cry2‐R amplify a 1.1‐kb product from the large toxin‐encoding plasmid (CP009999). Primers Cry1‐F and Cry1‐R amplify a 1.4‐kb product when any Cry1A gene is present; no amplification corresponds to loss of both *cry* gene‐bearing plasmids.

PCR used HotStar Taq (Qiagen) with cycling parameters: 5 min at 95°C, 30 × (45 s at 94°C, 1 min at 50°C [Cry2 primers] or 53°C [Cry1 primers], 90 s at 72°C), 10 min at 72°C. To provide DNA templates, fresh colonies were resuspended into 100 μl dH_2_O, frozen 20 min at −80°C, then boiled 10 min. Five microliters of supernatant was used in each 20 μl PCR mix. To exclude false‐negative results, each negative result was repeated three times from fresh colonies.

### 2.6 Whole‐genome sequencing

The unmarked ancestral *Bt kurstaki* 7.1.o strain was sequenced with PacBio after DNA extraction using the Qiagen high‐molecular weight kit. Data were produced using SMRTbell® Express Template Prep Kit 2.0 following the manufacturer's recommendations. This resulted in a 15‐ to 20‐kb insert library which was sequenced on a PacBio Sequel system using one cell per library and 10‐h sequencing movie time. The data were processed to provide CCS and single pass data and assembled using Unicycler version 0.4.7 (Wick et al., 2017).

For insect‐evolved lineages, the two clones with highest virulence based on a preliminary screening were chosen. At least two clones from each endpoint evolved lineage, in addition to the wt ancestor (7.1.o wt pHT315‐gfp) and the mutator (7.1.o ΔmutS), were sequenced. DNA was extracted using Qiagen DNeasy Blood and Tissue genomic extraction kits, after appropriate lysis for Gram‐positive bacteria. Sequencing was performed on an Illumina HiSeq 2500 at a minimum of 30× coverage with 125 bp paired‐end sequencing.

All Illumina reads were trimmed with Trimmomatic (Bolger et al., 2014). Unicycler version 0.4.7 was used to perform a hybrid assembly using PacBio self‐corrected reads and Illumina reads of the mutator ancestor (Wick et al., 2017). Since most evolutionary change occurred in the mutator lineages, the mutator ancestor provided a better reference for subsequent identification of mutations. This combination of read types resulted in a final assembly that included small plasmids that were otherwise lost from the PacBio data‐only assemblies, due to the DNA fragment size section being larger than the size of the plasmids. Short read data were used to validate and aid the comparison of plasmid genomes between a reference (Bt HD‐1) and Bt 7.1.o (this study) by mapping the short reads to the assembly (Li, 2012; Tables S2 and S3).

The final assembly was then checked against other *Bacillus thuringiensis kurstaki* strains using MAUVE (Darling et al., 2011) to check for possible genome errors. The assembled genome was then run though the software PROKKA (Seemann, 2014) providing a draft annotation. The PROKKA flag—use genus—genus *Bacillus* was used to improve taxon‐specific gene annotations. The final assembly was then checked against other Illumina whole‐genome shotgun data from the selection experiments. Sequenced clones and the final reference assembly were then analyzed with snippy version 4.4.0 (https://github.com/tseemann/snippy) to align reads against the newly assembled reference and used to call single‐nucleotide polymorphisms (SNPs). The Snippy SNP data were used to generate a Jukes–Cantor Neighbor‐Joining (1000 boot strap replicates) phylogeny of isolates, implemented with Geneious Prime 2022.2.1.

### 2.7 Fitness measurements

Measurements of relative fitness used a mutant of the 7.1.o isolate constructed by transformation with a pHT315‐rfp plasmid containing the *tetR* gene conferring tetracycline resistance (Zhou et al., 2014). This version of the ancestor, therefore, has a plasmid with a similar backbone to the evolved wild‐type clones but is distinguishable using its distinct antibiotic resistance. Relative fitness was calculated from an estimate of relative reproductive rates (Malthusian parameter) of the two genotypes as described previously (Zhou et al., 2020).

A 50/50 mix of spores of the two competitors was used to initiate competition experiments and was produced under standard conditions. Fitness was measured in conditions duplicating the selection experiment, that is, infection and transfer of cadaver to sporulating media, although insect cadavers were processed individually and HCO culture did not use antibiotics. After spore washing, mixes were plated on antibiotic‐free medium as well as tetracycline 10 μg/ml (for the standard competitor ancestor). The density of evolved clones in cadavers was calculated by subtracting competitor counts from antibiotic‐free plates total counts, as the *cat* gene present in the mut strain proved unreliable at allowing growth on chloramphenicol on LBA and we wished to use a common method for both wt and mut clones.

### 2.8 Assessment of toxin production

Bt bacteria were cultured in sporulation medium as above. Crystal protein production was assessed by centrifuging 1 ml of culture and resuspending in 1 ml of water, followed by two rounds of sonication, centrifugation, and washing to break open un‐lysed cells. The final pellet was resuspended in 1 ml of water. Serial dilutions were plated out in order to calculate the concentration of viable spores as CFUs. SDS‐PAGE analysis was then performed on equal numbers of CFUs to compare the production of crystal proteins. For the assessment of secreted Vip3A production, culture supernatant was passed through a 0.22‐μm cellulose acetate filter and to 1 ml of the filtrate 50 μl of 2% sodium deoxycholate was added and incubated for 30 min on ice. One hundred and fifty microliters of 100% Trichloroacetic acid was then added, the sample was vortexed and incubated overnight at 4°C. The precipitate was spun down at 15,000 *g* for 10 min, the supernatant removed, and the pellet was washed twice with ice cold 70% acetone then air‐dried. Following the addition of 20 μl water, the mixture was sonicated for 2 min to resuspend the pellet. For immunodetection of Vip3Aa, samples were boiled with SDS sample buffer and spotted onto a nitrocellulose membrane. A Vip3A antibody (a kind gift from Professor Juan Ferré) was used in association with an anti‐rabbit IgG HRP‐linked antibody for enhanced chemiluminescent detection.

### 2.9 Statistical analysis

All statistical analyses were carried out in R (v4.0.4) (https://www.R‐project.org). Bioassay and mortality data were analyzed using one of two methods. For comparisons between independently evolved lineages, we used generalized linear modeling (glms) with logit link functions and quasibinomial errors to correct for overdispersion. However, to test for treatment effects, or account for random effects associated with different lineages within treatments, we used generalized linear mixed models (glmer) in the package *lme4* (Bates et al., 2015). Although we attempted to use glmer models with binomial errors, these commonly failed to converge, especially in more complex models with dose × treatment interactions. Instead, we used the conservative approach of arc‐sine transforming proportional mortality and using normal errors. The glm and glmer approaches produce qualitatively similar results. Hypothesis testing in glmers used likelihood ratio tests after model simplification. LC_50_s and their standard errors were calculated using the *dose.p* function in the *MASS* package (Venables & Ripley, 2002) after fitting simple logit models using log_10_ dose and quasibinomial errors.

Analyses of viable spore production also used glmers with lineage fitted as a random effect, while the analysis of competitive fitness used evolved clone as a random effect and genotype (mutator or wild type) and number of toxins as explanatory variables. Model assumptions (normality, heteroskedasticity) were checked with graphical analyses and qq plots. Raw experimental data are available from Zenodo (Dimitriu et al., 2022).

## 3 RESULTS

### 3.1 Characterization of the mut ancestor

Wild‐type (wt) mutation rate toward rifampicin resistance was 7 × 10^−10^ [95% CI 4.2 × 10^−10^ to 1.02 × 10^−9^] while for the *ΔmutS* strain, the mutation rate was 1.76 × 10^−8^ [95% CI 1.48 × 10^−8^ to 2.08 × 10^−8^], showing a 25‐fold increase compared to the wt strain. The mutator, prior to passage, did not show any change in virulence relative to the ancestor (log_10_ LC50 = 4.9, test for difference from ancestor‐effect size 0.21, SE = 1.22, *p* = 0.87). The competitive fitness of the mutator strain under the conditions of the passage experiment also did not differ from the wt ancestor (*t* = 0.49, *p* = 0.63, means ± SE of mut and wt are 1.09 ± 0.06 and 1.13 ± 0.06, respectively).

### 3.2 Changes in virulence in evolved lineages

Duplicated endpoint assays of evolved lineages used total mortality, normalized to that of the wt ancestor in each experiment, to assess changes in virulence after five rounds of selection (ca. 60 generations). These assays showed that increased mutation rate and infectivity selection between subpopulations resulted in increases in normalized mortality relative to the in vitro controls (Figure 2a). Both strain (wild type or mutator) (*F*_1,44_ = 12.3, *p* = 0.001 at 3 days, *F*_1,44_ = 10.6, *p* = 0.002 at 5 days) and selection treatment (3 days, *F*_2,44_ = 21.4, *p* < 0.001; 5 days, *F*_2,44_ = 8, *p* = 0.0011) affected virulence of evolved lineages. Post hoc tests confirm increased virulence for infectivity versus yield selected treatments and for mutators versus wild‐type lineages (Tukey tests, all *p* < 0.01). Differences between treatments were larger when assessing early normalized mortality, suggesting changes in virulence affected timing and overall levels of mortality. We also used mixed models of arc‐sine transformed mortality, which fitted independent lineage as a random effect. These gave qualitatively similar results for day 3 and day 5 mortality, except in the mixed model analysis mutators and the infectivity treatment could be seen to increase virulence by causing more mortality at higher doses (dose × selection treatment interactions and dose × strain interactions, all *p* < 0.01 day 3, and *p* < 0.001, day 5 mortality).

Since the evolved lineages were not genetically homogeneous, we also conducted bioassays of six clones isolated from each lineage. Clonal assays of day 5 mortality show that mutation rate and infectivity selection increased virulence via interactions with dose (mixed effect *glm* with lineage as random effect: dose × selection treatment interaction df = 3, LR = 16.95, *p* < 0.001; dose*strain interaction df = 1, LR = 12.59, *p* < 0.001; Figures S1 and S2). We identified a number of clones with substantial increases in virulence, quantified as a reduction of LC50 of more than an order of magnitude from endpoint assays (Table S1).

### 3.3 Patterns of Cry toxin plasmid carriage

We saw variation between independent evolved lineages in terms of whether or not they increased or decreased virulence with respect to ancestors (Figure 2b, scatter plots). Low‐virulence lineages clearly had low mortality that did not increase with dose (glm lineage × dose interaction *F*_1,17_ = 5.92, *p* = 0.026). Cry toxins are encoded on two plasmids in Bt *kurstaki*—a mega‐plasmid containing Cry1Aa, Cry1Ac, and Cry2Aa, and a circa 80 kb plasmid‐carrying Cry1Ab (Méric et al., 2018). We used PCR primers to screen for loss of plasmids to test if loss of virulence in evolved lineages could be explained by changes in plasmid carriage. We observed numerous events of toxin gene loss in clones isolated from in vivo evolved lineages (Figure 2b bar plots) that correlated with loss of virulence. In particular, wild‐type lineages lost more plasmids than mutator lineages (Figure 2b; Fisher's exact test two‐tailed *p* = 0.006). Importantly, the infectivity selection regime was more effective at retaining these plasmids and preventing invasion of putative cheaters (Figure 2b, Fisher's exact test two‐tailed *p* = 0.00032).

### 3.4 Social cheating and Cry plasmid loss

**FIGURE 3:**
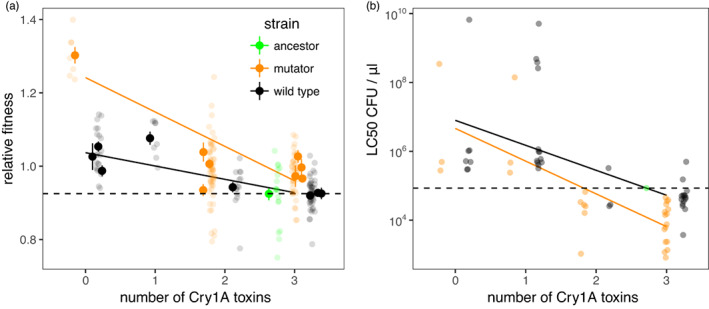
Social cheating and fitness in experimentally evolved Bt lines. Evolved clones that had lost virulence plasmids encoding Cry genes had higher competitive fitness (a) and lower virulence (b) than the ancestor. Toxin complements in A (*n* = 16) are derived from Illumina sequencing data; LC50s are shown for all evolved clones with toxin complement data based on PCR (*n* = 70). Means ± SE of fitness for each clone are plotted as solid points, raw data are plotted at 50% transparency, the dashed line is a reference line for the ancestor while solid lines show fitted statistical models (*glms* fitted with strain and number of Cry toxins).

Previously, we have seen that Cry toxin production is a public good and loss of investment in Cry toxins can be driven by the selective advantage of increased competitive growth rate within hosts, or social cheating. In order to test whether mutants that had lost Cry‐toxin plasmids were cheaters, we measured the fitness of one clone from each lineage in competition experiments that used a marked RFP mutant derived from the ancestor (Figure 3a). Mutants carrying fewer Cry toxin genes had higher competitive fitness (mixed model likelihood ratio test (LRT) df = 1, likelihood ratio (LR) = 9.05, *p* < 0.0026, Figure 3a). Mutants which had lost Cry toxins also had lower virulence, that is, higher LC_50_ (*glm F*_1, 64_ = 67.1, *p* < 0.0001, Figure 3b). Thus, these data were consistent with the hypothesis that low‐virulence mutants were free‐loading on investment in Cry toxins by other variants in their lineages (Raymond et al., 2012).

After accounting for toxin production, mutators evolved greater competitive fitness than wild‐type lineages (Figure 3a; mixed model, df = 1, LR = 7.5, *p =* 0.006) and had increased virulence (i.e., lower LC_50_s; *glm F*_1, 63_ = 7.35, *p* < 0.0157, Figure 3b) suggesting that increasing mutation rate increased the supply of beneficial alleles without reducing overall fitness. Since the original mutator mutant has indistinguishable fitness from the wild‐type ancestor (see Section 2, Methods), the initial genetic background of these strains does not explain this pattern.

### 3.5 Spore production

**FIGURE 4:**
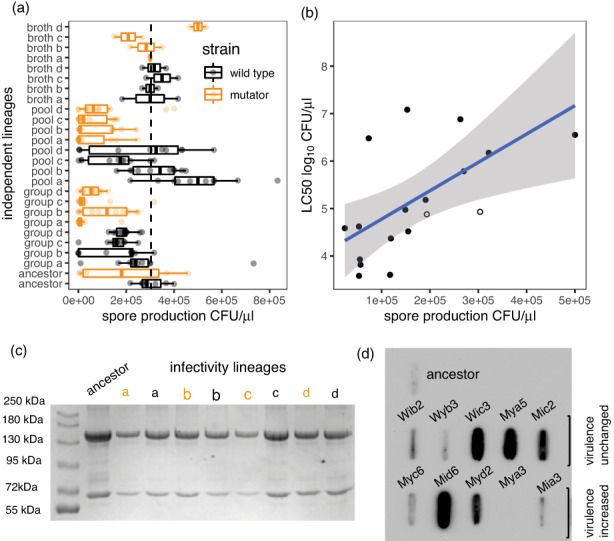
Increased virulence trade‐offs against reduced spore production in vitro in evolved Bt lines but is not associated with increased Vip3 production. (a) Variation in spore production between evolved lineages; asterisks indicate the significance of contrasts between all evolved lineages and the ancestor in a *glm*; boxplots summarize the results from two assays of each clone. (b) Mean LC50 of each in vivo passaged lineage (averaged across clones) plotted against spore production, ancestors plotted as hollow circles. (c) SDS PAGE showing Cry toxin production for all infectivity‐selected lineages, see Figure S3 for examples of individual clones. (d) Slot blot characterization of Vip3 production in the ancestral Bt clone and clones with increased virulence or virulence unchanged relative to the ancestor (see Table S1). See also Figures S3–S5 and Tables S2 and S3 for additional details on genomic and proteomic characterization of evolved clones.

The other major life‐history trait that we explored was the efficiency of spore production in the sporulation media used in the selection experiments. We saw lower spore production in the mutator and the infectivity‐selected lineages, the treatments that produced higher virulence (Figure 4a, mixed models, selection treatment df = 3, LR = 11.3, *p* = 0.01, mutation rate df = 1, LR = 8.36, *p* < 0.01). Importantly, between lineages, lower spore production was associated with lower LC_50_, which corresponds to higher virulence (Figure 4b, *F*_1,14_ = 10.5, *p* < 0.01, adj. *R* ^*2*^ = 0.39). We examined bacterial development and sporulation after 3 days (the typical growth period in sporulation media) and after 6 days for clones with elevated virulence (*n* = 3), the wild‐type ancestor and clones with unchanged virulence (*n* = 3). Elevated virulence was associated with delayed development. After 3 days, cultures of high‐virulence clones retained 5%–10% vegetative cells, while 85%–95% cells had completed development and undergone exosporium lysis. For clones with unchanged virulence, these figures were 1% vegetative cells and 99% lysis. After 6 days, all clones showed 98%–99% lysis indicating that clones with elevated virulence could eventually undergo complete sporulation.

This is biologically significant because toxin production is linked to sporulation in Bt, especially the Cry1A toxins that are the dominant virulence factors in our experimental strains (Deng et al., 2014). In addition, we selected for sporulation by heat‐treating preparations at the end of each round of selection to kill vegetative cells and possible contaminants. Reduced sporulation would therefore be expected to result in lower Cry toxin production and reduced virulence. Two classes of toxins are produced by Bt *kurstaki* earlier in the growth cycle than the Cry1A toxins and are expected to have activity against Cry1A‐resistant insects: These are the Cry2 toxins and the vegetative virulence factor Vip3Aa (Carrière et al., 2015; Deng et al., 2014). However, we found no significant differences in the ratio of Cry1A and Cry2 production that correlated with changes in virulence. At the lineage level, neither mutators nor infectivity‐selected replicates had elevated Cry2 production (Figure 4c), nor did we see differences in Cry2 production between the ancestor and evolved clones with high virulence (Figure S3). We did find substantial variation in secretion of Vip3Aa between evolved clones, but no association with increased virulence was seen (Figure 4d).

To seek mechanistic explanations for the reduced sporulation and increased virulence, the genomes of two clones from each lineage were sequenced and compared to a long‐read assembly of our ancestor. The ancestral genome consisted of a 5.7‐Mb chromosome and 14 plasmids that resolved as single contigs with high similarity to other Bt *kurstaki* genomes (Tables S2 and S3, Figure S4). Resequencing of evolved mutants confirmed that none carried mutations in their Cry genes or in regions immediately upstream. Comparison to the ancestral genome showed both small‐scale mutations and large‐scale deletions associated with plasmid loss. Particularly, toxin gene loss patterns detected previously by PCR can be explained by the loss of one or both Cry plasmids.

**FIGURE 5:**
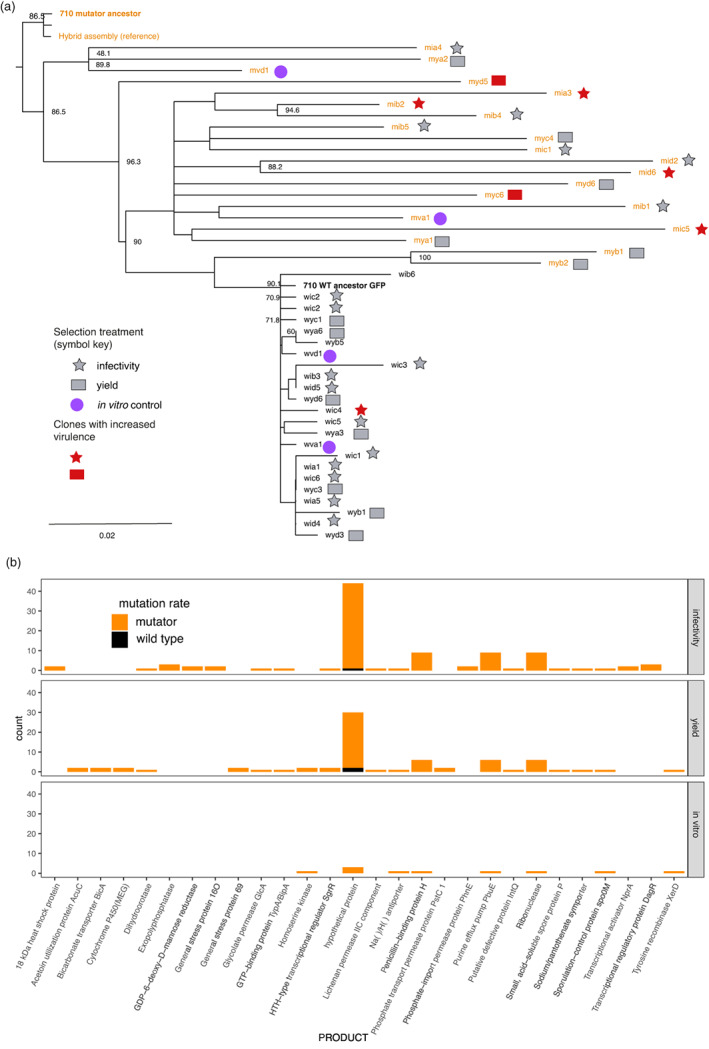
Genomic analyses of evolved Bt clones. (a) Consensus neighbor‐joining tree of sequenced evolved clones. For ease of interpretation, bootstrap of values >50 only has been displayed for the nodes. The scale bar indicates the number of substitutions per nucleotide. The reference refers to the hybrid PacBio/Illumina assembly, all other sequence information is derived from SNPs identified using Illumina data with reference to this assembly. (b) Histogram of nonsynonymous mutations identified in at least two evolved clones in our three selection regimes, infectivity selection, yield selection, and the in vitro passage controls.

**FIGURE 6:**
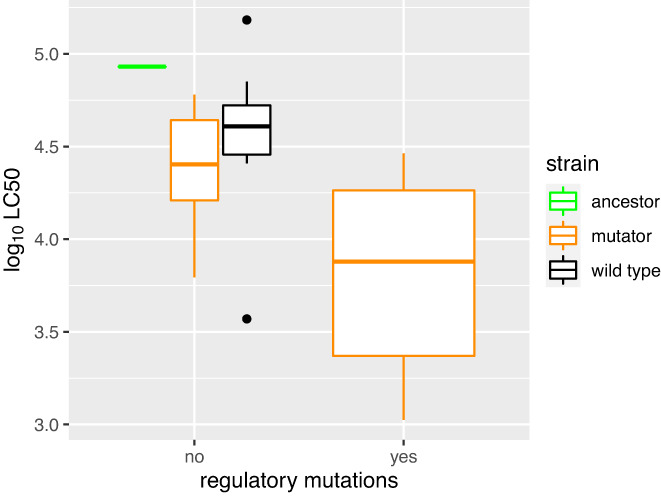
Virulence of evolved mutators clones with and without loss of function mutations in putative transcriptional regulators. Virulence is expressed as log LC_50_ with evolved wild‐type clones and the 7.1.o ancestor plotted as comparisons, after excluding data from sequenced clones that are Cry toxin cheaters (i.e., which carry 0 or 1 Cry toxin genes). Table S4 contains a list of mutations

Phylogenetic analysis of our evolved clones shows that there was considerably more genome evolution in the mutators (Figure 5a). However, evolved clones with similar virulence phenotypes did not group together, nor did clones from the same treatments (Figure 5a). Mapping of mutations to the reference also suggests minimal convergent evolution in terms of shared changes in DNA (although there is phenotypic convergent evolution in terms of sporulation). We did identify a small list of genes that acquired mutations in more than one clone (Figure 5b); this list includes three transcriptional regulators *nprA* (in clones Mia3 and Mia4), *dagR*, and *sgrR* with nonsynonymous mutations in insect‐passaged lineage, but not in the in vitro controls. In general, missense or frameshift mutations were prevalent in genes encoding putative transcriptional regulators (Table S4). These included well‐described regulators such as NprA but also proteins with helix–turn–helix domains. Mutator clones from the infectivity selection treatment accounted for 10 of the 16 loss of function mutations in putative transcriptional regulators. If we compared the virulence (LC_50_) of all the sequenced mutator clones, there was a significant increase in virulence in the mutator clones with these loss of function mutations compared to those with no mutations in regulatory genes (*F*_1,16_ = 5.91, *p* = 0.027, Figure 6), assuming that the data from these clones are independent observations.

## 4 DISCUSSION

Kin selection theory emphasizes that altered virulence can have different impacts on individual‐ and group‐level fitness components (Buckling & Brockhurst, 2008). Notably, investment in bacterial virulence factors requires resources to be diverted away from growth of individual cells. If virulence factors are diffusible, they may behave as “public goods” by conferring fitness benefits that are shared among members of an infecting group (Diard, Sellin, et al., 2014; Harrison et al., 2006; Raymond et al., 2012). If cells reduce investment in public goods virulence, these “cheaters” can gain a reproductive advantage during growth in the host by freeloading on the products of others (Buckling & Brockhurst, 2008; Diggle et al., 2007; West & Buckling, 2003), although this may be accompanied by a group‐level cost such as reduced infectivity. Virulence factors can behave as public goods both in laboratory models and infections of insects with biocontrol agents such as entomopathogenic nematodes and Bt (Deng et al., 2015; Raymond et al., 2012; Shapiro‐Ilan & Raymond, 2016; Zhou et al., 2014). In this experiment, it was clear that some clones within lineages had reduced virulence relative to the ancestor and so were putative cheaters and we identified a link between the absence of Cry toxins and increases in competitive fitness.

This result mirrors previous experiments with Bt strains that have been cured of Cry toxin‐encoding plasmids as well as competition between Bt and naturally Cry null *B. cereus* (Raymond et al., 2007, 2012). The key difference in this study is that loss of Cry toxin plasmids occurred spontaneously during experimental evolution in multiple independent lineages and in different ways, that is, by the loss of either or both of the large plasmids that encode these toxins. In addition, the selection pressure we imposed during experimental evolution affected how readily Cry toxins were lost: selection pressure to maximize yield (final bacterial population size in cadavers) resulted in higher rates of plasmid loss than in the infectivity selection treatment. The loss of Cry plasmids in response to selection on yield occurred because Cry toxins impose costs on growth rate *and* total production of spores within hosts (Raymond et al., 2012). Both social evolution and evolution of virulence theory commonly divide the selective forces into individual (within‐host)‐ and group‐level (between‐hosts) components; these are often in conflict in microbes (Cressler et al., 2016; Frank, 1997). Other bacterial virulence factors which are public goods, such as siderophores, have a group‐level benefit in terms of increasing yield within the host (Harrison et al., 2006; West & Buckling, 2003). However, Cry toxins are produced at sporulation in the cadaver, rather than in the early stages of infection, and these Cry toxins require so much protein production that Bt variants which invest in these toxins produce substantially fewer spores per unit resource than their Cry null counterparts (Deng et al., 2015; Raymond et al., 2012). In essence, Cry toxins are a different type of public good than siderophores and act to increase infectivity rather than the quality of resources available for growth within hosts. It remains to be seen whether other bacterial public goods can also reduce the population size of pathogens in hosts but increase infectivity or transmission.

The infectivity selection was not only more effective at retaining Cry plasmids but also resulted in greatest gains in virulence relative to the ancestor. There are several factors that could have contributed to these results. First, budding dispersal in a metapopulation involved less pooling of cadavers than the yield selection treatment. Since individual cadavers are likely to be dominated by different genotypes (van Leeuwen et al., 2015), pooling cadavers can reduce relatedness relative to infectivity selection treatment. In other words, there is more scope for cheating and greater selection to increase competitive fitness in the yield treatment. An additional consideration is that the infectivity selection also imposed selection for gains in virulence at an additional spatial scale. In all treatments, there was selection for infectivity at a between‐host level (successful infections had to occur in order for passage to proceed), but by using selection in a metapopulation, we imposed an additional level of competition between subpopulations within a metapopulation. The effects of this level of selection for infectivity in pathogens require further investigation, but we would hypothesize that competition between subpopulations is more important when investment in virulence has costs in terms of growth rate and population size within hosts (Raymond & Erdos, 2022). The primary aim of this study was to devise and test different methods of selection for maintaining and improving virulence, and so we will require further work to tease out the precise evolutionary mechanisms behind the success of this particular treatment.

The mutator lines showed clear differences in the rate of molecular evolution as well as greater increases in virulence relative to the wild‐type background. In contrast to previous work, we did not see an increase in levels of cheating under high mutation rate (Harrison & Buckling, 2005; Racey et al., 2009). Mutagenesis is very commonly employed in strain improvement and directed evolution with *Bacillus* sp. and other microbes (Lai et al., 2004; Raju & Divakar, 2013). The evidence for the ability of mutators to accelerate increases in fitness is complex and has several unresolved questions. Mutators can increase the supply of both beneficial and deleterious alleles and it is clear that there is no known direct benefit to having impaired proofreading: mutator alleles rise in frequency by hitchhiking on the fitness benefits of linked alleles (Chao & Cox, 1983; de Visser, 2002; Gentile et al., 2011; Raynes et al., 2013). Mutators are often seen at quite high proportions in pathogenic bacteria (LeClerc et al., 1996; Matic et al., 1997; Oliver et al., 2000) and mutators can appear spontaneously in experimental evolution although evolving lineages often moderate mutation rates during long‐term transfer experiments (Good et al., 2017; Ho et al., 2021).

One reason for the prevalence of mutators among pathogens, and for their success in this study is that small populations and/or infection bottlenecks may limit the supply of beneficial mutations (de Visser, 2002). While demographics and mutation supply are known to affect the long‐term success of mutators, simulation and experimental studies suggest that bottlenecks or small populations may not favor high mutation rates (Ho et al., 2021; Raynes et al., 2018). Nevertheless, mutators are more successful when large‐effect beneficial mutations are available for hitchhiking (Gentile et al., 2011; Mao et al., 1997; Thompson et al., 2006) and the strong selection imposed by new sporulation conditions and a novel host genotype in this study may account for the success of mutators in our experiments. Overall, the use of mutators to overcome host resistance has a sensible biological basis. However, this may have had implications for the stability of virulence (Figure S2). Potentially more stable phenotypes can be produced by periodic rounds of chemically induced mutagenesis or by using initial pathogen populations with high standing genetic variation.

It was not possible to identify a single simple cause for any gains in virulence in this study, although this was consistently related to decreased sporulation efficiency. Reduced sporulation means that vegetative cells and their secreted proteins will be more prevalent in final inocula. We observed that a proportion of mutants with reduced sporulation gave increased virulence per spore but not in terms of dilution of the broth in which they were cultured. This is potentially because standardizing doses by spore means that a higher concentration of broth‐associated virulence factors are included in inocula. As yet, none of the classic virulence factors associated with broth such as Vip3A appear to be responsible for this increase in virulence. There are many virulence factors secreted into broth by Bt and its relatives (Chitlaru et al., 2006; Guinebretière et al., 2002; Stenfors Arnesen et al., 2008). While spores and crystals are by far the most significant contributors to virulence in Bt (Raymond et al., 2010), broth‐associated virulence may be more significant for Cry1A‐resistant hosts and are known to be significant for some hosts such as *Galleria mellonella* (Salamitou et al., 2000).

For example, secreted virulence factors are regulated by a quorum‐sensing system (PlcR/PapR) that activates a suite of virulence factors involved in host invasion (Salamitou et al., 2000; Zhou et al., 2014). Although these are typically produced at stationary phase, expression of the PlcR‐regulated genes is repressed in low‐nutrient sporulation medium such as HCO (Lereclus et al., 2000). The stationary phase secretome of Bt and *B. anthracis* in sporulation medium is mainly composed of metalloproteases such as Inh1A and NprA and is much reduced in the diversity of proteins compared to nutrient‐rich media (Chitlaru et al., 2006; Perchat et al., 2011). These metalloproteases are hypothesized to have a role in the digestion of cadavers in late‐stage infections (Perchat et al., 2016). Nevertheless, they are essentially lytic enzymes and could improve the ability of Bt to invade the host at high concentration. Other possible virulence factors secreted into sporulating media include chitinase‐ and chitin‐binding proteins (Chitlaru et al., 2006). Notably, we did identify mutations in *nprA* and other transcriptional regulators in several insect‐passaged mutators.

Bt is an important pathogen for both organic horticulture and modern “biotech” crops, so the question of how to improve strains or discover new toxins is significant, especially given the need to respond to the evolution of resistance (Adang et al., 2014; Badran et al., 2016). However, these methods may be applicable to other parasites, such as nematodes and fungi, which also produce costly excreted virulence factors (Shapiro‐Ilan & Raymond, 2016). Moreover, in addition to providing a framework for increasing virulence, the combination of in vivo and in vitro selection used here could be applied when the aim is adaptation to an alternative growth medium or the improvement of other phenotypes, while retaining insecticidal efficacy. For some pathogens, for example, fungi, it is also worth bearing in mind that while virulence traits may not be social (there are no data yet), other important biocontrol traits such as sporulation are likely to be subject to social conflicts. The timing of a switch to any resting state (spore, persister, etc.) can be subject to cheating—for example, cessation of growth can be cooperative and late switching to sporulation can provide growth advantages in competition (Gardner et al., 2007; Ratcliff et al., 2013). Efficient sporulation is essential for the production of both fungal and bacterial biocontrol agents and can be readily lost during laboratory selection. In general, passage regimes that minimize social conflict and which can maintain valuable cooperative traits could have broad importance across diverse groups of invertebrate pathogens, and here, we have shown the value of a metapopulation regime that can reduce social conflicts. Moreover, experimental evolution offers a mechanism‐free solution to overcoming pest resistance which is potentially applicable to many pathogens and pests. In vivo selection experiment may help us discover entirely novel virulence factors or virulence modification responses, in contrast to directed evolution methods that require well‐understood protein–protein interactions (Badran et al., 2016).

## CONFLICT OF INTEREST

This work formed part of international patent application number WO 2019/030529 A1 by BR & NC.

## Supporting information


AppendixS1

## Data Availability

Sequence data are hosted at the SRA under BIOPROJECT SUB10359598. Plasmids, ancestor, and mutator clones are available on request. Evolved mutants can be shared subject to Material Transfer Agreements. Experimental data are available at Zenodo with a permanent doi: 10.5281/zenodo.7503995.
