# Supplementary material for: Selecting for infectivity across metapopulations can increase virulence in the social microbe Bacillus thuringiensis
Source: Evol Appl. 2023 Jan 16;16(3):705–20. doi: 10.1111/eva.13529 (PMC10033855; doi:10.1111/eva.13529)
Supplement: Supplementary file 1 — AppendixS1 [file EVA-16-705-s001.pdf]

Figure S1. Variation in spore associated virulence across evolved *in vivo* clones. Logit-transformed mortality 5 days after inoculation is shown as a function of Bt dose as $\log_{10}$ heat tolerant CFU per μl (**A**) and as a volume of standard diluted inoculant instead of dose (**B**), to avoid false positives arising from low plating efficiency/very low sporulation for some clones. The black line in each panel describes the dose response for the ancestor (from a glm with quasibinomial errors). Columns a-d refer to the independently evolved lineages.

A

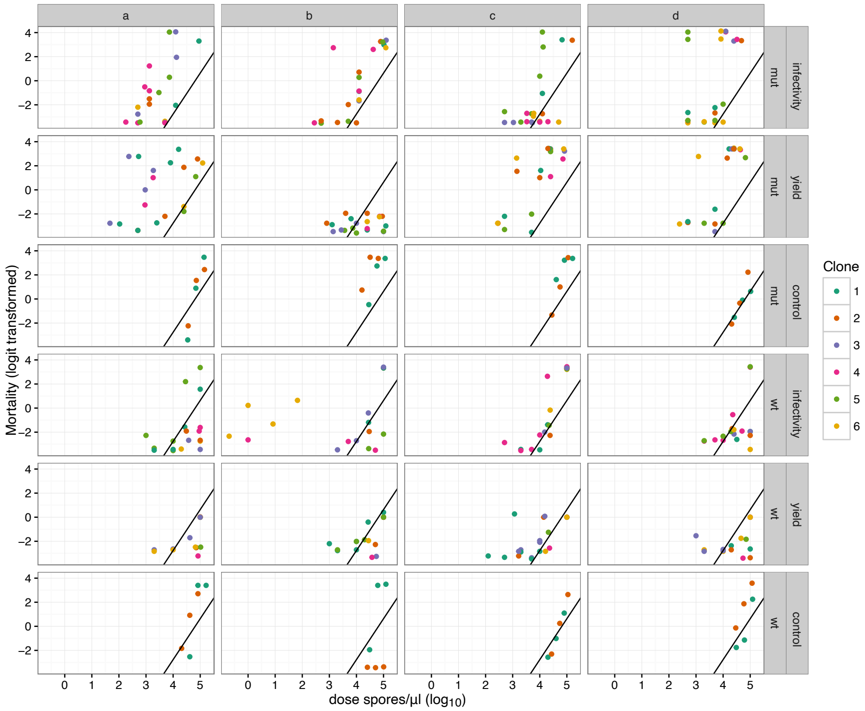

B

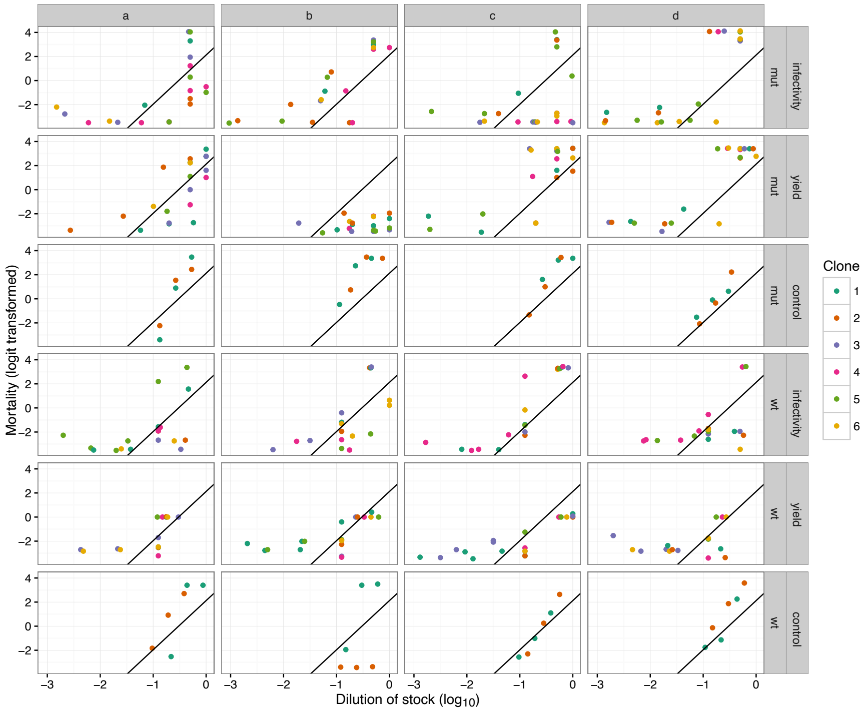

Figure S2. Comparison of the virulence of 5th round evolved whole populations and clonal isolates. Logit-transformed mortality 5 days after inoculation is shown as a function of Bt dose. Bt dose is shown as volume of standard diluted inoculant instead of dose (CFUs), to avoid false positives arising from low plating efficiency for some clones. Clone data outside of the range of doses tested for population assays were excluded. Virulence of mutator (mut) populations was significantly higher than that of mut clones, whereas virulence of wild type (wt) populations was not different from that of wild type clones (post-hoc Tukey tests on logit-transformed mortality ~ dose * treatment * clone:strain, mut mix > mut clones, z=10.9, p<0.001; wt mix < wt clones, z=-0.93, p=0.78) suggesting that mutators had reduced phenotypic stability relative to wt clones.

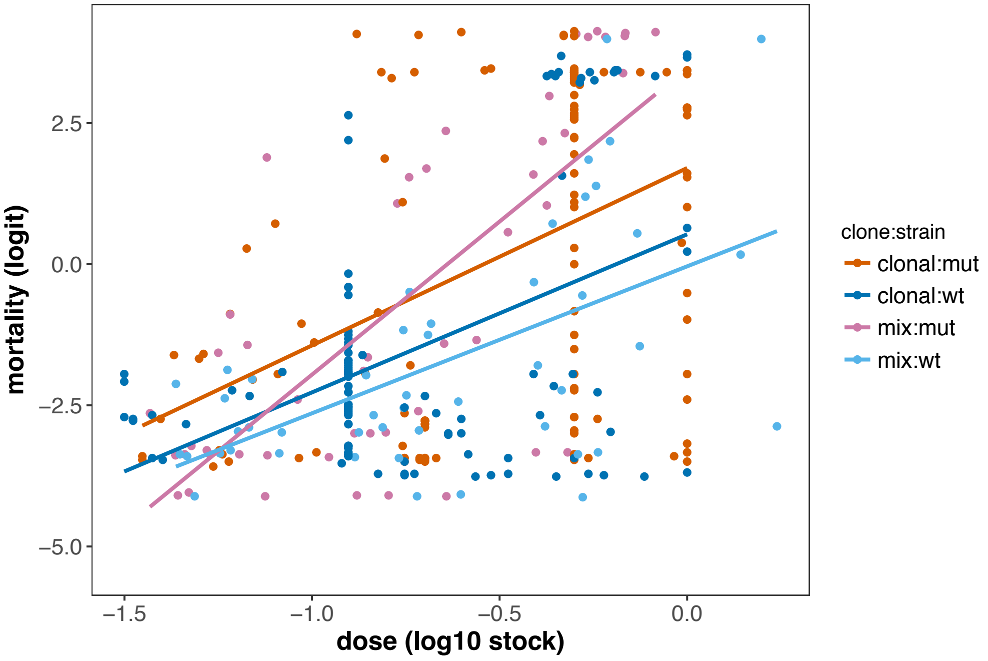

Figure S3. SDS PAGE gels showing the relative production of Cry1 and Cry2 toxins (at approximately 140 and 70 kDa respectively) by the Bt *kurstaki* ancestor and two evolved clones. Mia3 and Myc6 both showed increases in virulence relative to the ancestor (see Table S1). Culture methods replicated those in the selection experiment (three days in HCO); equal culture volumes were loaded in this gel.

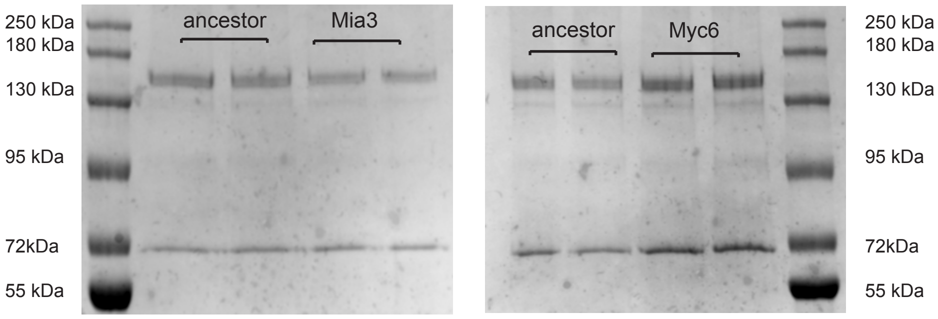

Figure S4. Comparison of plasmid genomes between the reference strain HD1 and the 71o strain (ancestor for this study). The graphic is to scale and based on a Mauve (https://doi.org/10.1371/journal.pone.0011147) output annotated to better show inverted, unique regions and missing plasmids between the two strains. The blocks of colour and the grey joining ribbons correspond to homologous regions between the strain plasmids. Plasmid numbers are matched to reference accession in Table S2**.**

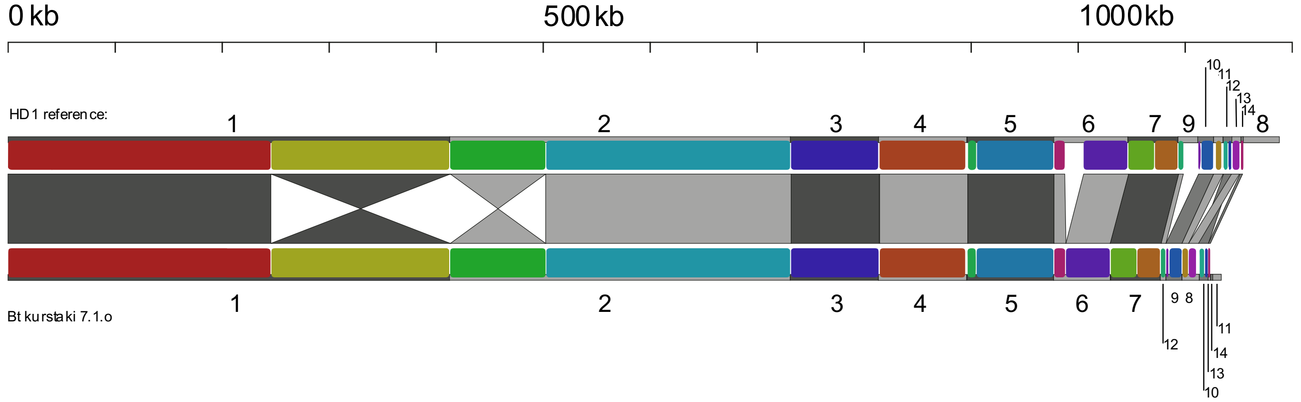

Table S1 Virulence (LC50) of selected clones from the passage experiment. LC50 concentrations are in $\log_{10}$ CFU μL^{-1}. Changes in virulence with respect to ancestor were first tested in clone level analyses of all bioassay data using glms and quasibinomial doses with clone and $\log_{10}$ CFU μl^{-1} fitted as main effects. A subset of clones with putative increased virulence were re-assayed; differences in virulence with respect to the ancestor in post-hoc treatment contrasts are identified in this table (Significance of tests: * $p < 0.05$, ** $p < 0.01$; *** $p < 0.001$). For a subset of clones with increased virulence we present LC50s and their standard errors calculated using the *dose.p* function using the *MASS* package after fitting simple logit models using $\log_{10}$ dose and quasibinomial errors. We also list additional clones used in the proteomic analysis in Figure 4 C, D & E which did not increase virulence during passage: NS indicates a LC50 that is not significantly different from the ancestor. †These clones have also been assayed in VLSS insects (Bt susceptible) and show no loss of virulence in that host.

Clone	*Genetic Background*	*Log_{10} LC50* (SE). NA indicates indistinguishable from ancestor	*Increase in virulence relative to ancestor*
ancestor	wild-type	5.00 (0.1)	-
Mia3	mutator	3.82 (0.13)	15.1 ***
Mib2	mutator	4.21 (0.08)	6.2 ***
Mic5	mutator	3.99 (0.4)	10.2 ***
Mid6†	mutator	3.69 (0.3)	20.4 ***
Mya3	mutator	4.4 (0.2)	3.1 *
Myc6	mutator	3.9 (13.2)	11.2 **
Myd5†	mutator	4.24 (0.36)	5.8 **
Wic4†	wild type	4.44 (0.09)	3.6 ***
Mic2	mutator	NA	NS $p = 0.57$
Mya5	mutator	NA	NS $p = 0.44$
Wib2	wild-type	NA	NS $p = 0.76$
Wic3	wild-type	NA	NS $p = 0.47$
Wyb3	wild-type	NA	NS $p = 0.33$

Table S2. Summary of plasmid comparison between reference strain HD1 and the 71o strain.

Current study: strain 71o				HD1 reference			
**Contig**	**Plasmid no.**	**Length (bp)**	**Depth relative to chromosome**	**Plasmid no.**	**HD1 accession**	**Length (bp)**	**Size difference (bp)**
Chromosome 1	NA	5,655,593	1.00x	na	CP010005	5,675,189	(19,596)
2	1	412,028	2.08x	1	CP009998	412,022	6
3	2	317,339	4.43x	2	CP009999	317,336	3
4	3	82,377	2.21x	3	CP010000	82,528	(151)
5	4	82,304	2.88x	4	CP010001	82,303	1
6	5	80,699	1.15x	5	CP010002	80,285	414
7	6	52,548	4.20x	6	CP010003	69,317	(16,769)
8	7	46,634	10.19x	7	CP010006	46,634	-
9	8	16,148	26.38x	12	CP010007	8,513	7,635
10	9	14,889	4.65x	10	CP010010	14,889	-
11	10	8,279	10.69x	11, 13	CP010009, CP010011	15,914	(7,635)
12	11	7,705	17.77x	no match	no match		7,705
13	12	5,113	14.80x	9	CP010012	18,332	(13,219)
14	13	2,062	23.33x	14	CP010008	2,062	-
15	14	1,944	11.34x	no match	no match		1,944
Total (bp)		6,785,662				6,825,324	73812

Table S3. Coverage of 71o strain short read sequence data mapping to the HD1 reference strain. Confirming the absence of HD1 plasmid CP010004 in the 71o genome.

HD1 accession	Plasmid number	HD1 reference size	number of short reads mapping	coverage	copy number vs chromosome
CP010005	Chromosome	5,675,189	3,054,392	48	1.0
CP009998	1	412,022	442,079	97	2.0
CP009999	2	317,336	22,100	205	4.2
CP010000	3	82,528	111,588	122	2.5
CP010001	4	82,303	132,927	145	3.0
CP010002	5	80,285	118,666	133	2.7
CP010003	6	69,317	166,436	216	4.5
CP010006	7	46,634	270,602	522	10.8
CP010004	8	34,150			
CP010012	9	18,331	208,629	1,024	21.1
CP010010	10	14,889	39,498	239	4.9
CP010007	11	8,513	152,832	1,616	33.4
CP010009	12	8,279	62,718	682	14.1
CP010011	13	7,635	78,095	921	19.0
CP010008	14	2,062	27,347	1,194	24.6

Table S4. Passaged clones with mutations in putative transcriptional regulators. HTH denotes Helix turn Helix proteins.

Background	Selection treatment	Lineage	Clone	Protein	Type of mutation
Mutator	Infectivity	mga	3	NprA	stop gained
Mutator	Infectivity	mga	4	NprA	frameshift
Mutator	Infectivity	mgb	2	DagR	missense
Mutator	Infectivity	mgb	4	DagR	missense
Mutator	Infectivity	mgb	4	repressor PagR	missense
Mutator	Infectivity	mgc	1	DagR	missense
Mutator	Infectivity	mgc	1	LiaR	missense
Mutator	Infectivity	mgc	5	HTH-type regulator SgrR	missense
Mutator	Infectivity	mgc	5	HTH-type repressor KstR2	missense
Mutator	Infectivity	mgd	2	HTH-type regulator	frameshift
Mutator	Infectivity	mgd	2	activator HcaR	frameshift
Mutator	Yield	mpa	1	HTH regulator ImmR	missense
Mutator	Yield	mpa	2	LiaR	missense
Mutator	Yield	mpa	2	HTH-type regulator SgrR	frameshift
Mutator	Yield	mpc	6	HTH-regulator LutR	frameshift
Mutator	Yield	mpd	6	HTH-regulator NorG	missense
Mutator	*in vitro*	mva	1	HTH-regulator CysL	missense
		Synonymous mutations			
Mutator	*in vitro*	mvd	1	HTH-regulator CysL	synonymous
Mutator	*in vitro*	mvd	1	HTH-regulator CatM	synonymous
Mutator	Infectivity	mga	4	HTH-repressor ComR	synonymous
